# Low sulfated heparins target multiple proteins for central nervous system repair

**DOI:** 10.1002/glia.23562

**Published:** 2018-12-26

**Authors:** George A. McCanney, Michael A. McGrath, Thomas D. Otto, Richard Burchmore, Edwin A. Yates, Charles D. Bavington, Hugh J. Willison, Jeremy E. Turnbull, Susan C. Barnett

**Affiliations:** ^1^ Institute of Infection, Immunity, and Inflammation, College of Medical, Veterinary and Life Sciences, University of Glasgow Glasgow UK; ^2^ GlycoMar Limited, European Centre for Marine Biotechnology, Dunstaffnage Marine Laboratory Oban Argyll Scotland, UK; ^3^ Department of Biochemistry Institute of Integrative Biology, University of Liverpool Liverpool UK

**Keywords:** amyloid‐β, heparan sulfates, myelination, neurite outgrowth

## Abstract

The lack of endogenous repair following spinal cord injury (SCI) accounts for the frequent permanent deficits for which effective treatments are absent. Previously, we demonstrated that low sulfated modified heparin mimetics (LS‐mHeps) attenuate astrocytosis, suggesting they may represent a novel therapeutic approach. mHeps are glycomolecules with structural similarities to resident heparan sulfates (HS), which modulate cell signaling by both sequestering ligands, and acting as cofactors in the formation of ligand–receptor complexes. To explore whether mHeps can affect the myelination and neurite outgrowth necessary for repair after SCI, we created lesioned or demyelinated neural cell co‐cultures and exposed them with a panel of mHeps with varying degrees and positions of their sulfate moieties. LS‐mHep7 enhanced neurite outgrowth and myelination, whereas highly sulfated mHeps (HS‐mHeps) had attenuating effects. LS‐mHeps had no effects on myelination or neurite extension in developing, uninjured myelinating cultures, suggesting they might exert their proregenerating effects by modulating or sequestering inhibitory factors secreted after injury. To investigate this, we examined conditioned media from cultures using chemokine arrays and conducted an unbiased proteomics approach by applying TMT‐LC/MS to mHep7 affinity purified conditioned media from these cultures. Multiple protein factors reported to play a role in damage or repair mechanisms were identified, including amyloid betaA4. Amyloid beta peptide (1–42) was validated as an important candidate by treating myelination cultures and shown to inhibit myelination. Thus, we propose that LS‐mHeps exert multiple beneficial effects on mechanisms supporting enhanced repair, and represent novel candidates as therapeutics for CNS damage.

## 1 INTRODUCTION

Spinal cord injury (SCI) is predominantly caused by mechanical and ischemic trauma to the spinal cord. As the CNS has limited regenerative and repair capacity, any function loss is generally permanent. The pathophysiology of SCI comprises both a primary and secondary cascade of injury mechanisms (Dumont et al., 2001). The former causes axonal transection, cellular damage, and disruption of the blood‐brain barrier (BBB) while the latter leads to local neuronal cell death and Wallerian degeneration, accompanied by the initiation of an intense immune response. The CNS responds to these events by forming a glial scar rich in reactive astrocytes, myelin‐associated inhibitors, microglia, macrophages, and meningeal fibroblasts which play a major role in sequestering damaged tissue in a relatively impermeant environment (Filous & Silver, 2016). Thus, any repair strategies for SCI must consider multifactorial pathways including disruption of the scar, promotion of axonal outgrowth, and remyelination.

Previously, we used a confrontation assay to study the effects of astrocytosis, characterizing boundary formation, expression of GFAP, and astrocyte hypertrophy (Lakatos, Franklin, & Barnett, 2000; Wilby et al., 1999). We found that Schwann cells do not mingle with astrocytes whereas olfactory ensheathing cells (OECs) mingle well (Lakatos et al., 2000). We identified heparin and FGFs as strong inducers of boundary formation between astrocytes and OECs and concluded that highly sulfated heparan sulfates (HS) secreted by Schwann cells induced the astrocyte‐Schwann cell boundary (Fairless, Frame, & Barnett, 2005; Santos‐Silva et al., 2007).

HS are linear sulfated polysaccharides that play an important role in regulating many mammalian cellular processes. They exist as proteoglycans (HSPG) in which two or three HS chains are attached to core proteins at the cell surface, or extracellular matrix (ECM) proteins. It is thought that the pattern, epimerization, and degree of sulfation is important in facilitating HS–ligand interactions, thereby enabling HS to function as a key regulator of complex cell signaling mechanisms in development, regulation of chemokine function, angiogenesis, and blood coagulation, in both normal and disease states (Bishop, Schuksz, & Esko, 2007; Changyaleket, Deliu, Chignalia, & Feinstein, 2017; Cui, Freeman, Jacobson, & Small, 2013). To investigate how HS sulfation characteristics influence astrocytosis, we applied a panel of modified heparins (mHeps) which have been selectively desulfated to the confrontation assay. We found that mHeps with low sulfated (LS‐) moieties could attenuate the astrocytic behavior, whereas highly sulfated (HS‐) mHeps induced a strong reactive astrocyte response in culture (Higginson et al., 2012). This led us to hypothesize that modifying the level of HS sulfation in the vicinity of astrocytes might be a therapeutic approach to promoting one pathway involved in CNS repair (O'Neill et al., 2017).

Although the above addresses effects on astrocytosis, if mHeps are to be developed for treatment of the injured CNS, there is a need to investigate possible effects on other neural cells. Therefore, we studied the effect of mHeps on myelinating cultures generated from dissociated spinal cord cells. These basic cultures are referred to as myelinating cultures‐development (MC‐Dev). Second, an adaptation of MC‐Dev can be used to model aspects of CNS injury by cutting mature cultures with a scalpel blade, generating a cell‐free area characterized by a persistent lack of neurite outgrowth. Over time, the cell‐free area becomes infiltrated with reactive astrocytes, and microglia, as seen in animal models of CNS injury. These cultures are termed MC‐Inj (Boomkamp, Riehle, Wood, Olson, & Barnett, 2012). Finally, mature myelinating cultures can be used to investigate demyelination by treating them with anti‐MOG antibody plus complement which resulted in demyelination of axons (Elliott et al., 2012; MC‐Demy). When the panel of differentially sulfated mHeps was applied to these three cultures types, we found that LS‐mHeps not only attenuate astrocytosis but also exert positive effects on neurite outgrowth and remyelination suggesting these glycomolecules may be an important novel therapeutic for CNS damage. Using tandem mass tag (TMT) labelling and liquid chromatography–mass spectrometry (LC‐MS) and chemokine array of secreted factors from the two injury culture models, we identified differences in heparin binding proteins (HBPs) suggesting subtle difference in mechanisms.

## 2 MATERIALS AND METHODS

### 2.1 Astrocytes derived from neurospheres

Neurospheres (NS) were generated from the striata of 1‐day‐old Sprague–Dawley (SD) rat using a method modified by Reynolds and Weiss (1996) and differentiated into astrocytes as described in Sorensen, Moffat, Thomson, and Barnett (2008). Briefly, the tissue was enzymatically dissociated and plated in NS medium (NSM) containing, Dulbecco's Modified Eagle Medium/F12 (DMEM/F12, 1:1, DMEM containing 4,500 mg/L glucose, Life Technologies, Carlsbad, CA), enriched with 0.105% NaHCO_3_, l‐glutamine, 5,000 IU/ml penicillin, 5 μg/ml streptomycin, 5.0 mM HEPES (all from Invitrogen, UK), 100 μg/ml apotransferrin, 25 μg/ml insulin, 60 μM putrescine, 20 μM progesterone, and 30 μM sodium selenite, supplemented with 20 ng/ml of epithelial growth factor (EGF; all from Peprotech, UK). The cell suspension was maintained until NS were formed. To generate astrocytes, the NS were triturated to produce smaller cell sphere suspensions, transferred to 13 mm poly‐l‐lysine (PLL; 13 μg/ml, Sigma) coated coverslips in a 24‐well plate (Corning, UK) and incubated for a further 5–7 days in vitro (DIV) at 37°C in an atmosphere of 7% CO_2_/93% air until a confluent monolayer formed. NS‐derived astrocytes were maintained in DMEM‐1 g/ml glucose (Life Technologies) with 10% fetal bovine serum (Sigma, Poole, Dorset, UK) and 2 mM l‐glutamine (Sigma).

### 2.2 Modified heparins

Selectively chemically desulfated mHeps were prepared as described previously (Higginson et al., 2012) and illustrated in Table 1. The compounds described here had predominant repeating structures as follows (LS, low sulfated; HS, high sulfated): HS‐mHep 1, IdoA(2S)‐GlcNS(6S); LS‐mHep6, IdoA(2S)‐GlcNAc; LS‐mHep7, IdoA‐GlcNS; LS‐mHep8, IdoA‐GlcNAc.

**Table 1:** Library of modified heparins (mHeps): List of mHeps showing their predominant disaccharide repeat along with the corresponding chemical modification; (a) depicts fisher projection of commercial heparin; and (b) schematic of hexose sugar ring backbone with the carbons labeled

mHep	Disaccharide repeat	Modification
1	IdoA(2S)‐GlcNS(6S)	Heparin control
6	IdoA(2S)‐GlcNAc	6‐O‐desulfation/N‐acetlyation
7	IdoA‐GlcNS	2‐O‐desulfation /6‐O‐desulfation
8	IdoA‐GlcNAc	2‐O‐desulfation /6‐O‐desulfation /N‐acetlyation
(a) 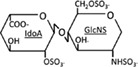		(b) 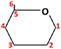

### 2.3 Myelinating spinal cord cultures (MC‐Dev and MC‐Inj)

Generation of rat spinal cord mixed cell cultures (myelinating cultures [MC]) was based on our previously described methods (Boomkamp et al., 2012; Boomkamp, McGrath, Houslay, & Barnett, 2014; Sorensen et al., 2008). The spinal cord of E15.5 SD embryos were enzymatically dissociated and the resulting cell suspension at 300,000 cells per 50 μl, were plated on top of the NS derived astrocytes on coverslips in plating medium (PM) that contained 50% DMEM‐1 g/ml glucose, 25% horse serum (Invitrogen), 25% HBSS (with Ca^2+^ and Mg^2+^, Life Technologies) and 2 mM l‐glutamine. Cells were left to adhere for 2 hr at 37°C, then supplemented with 300 μl PM and 500 μl differentiation medium which contained DMEM (4.5 g/ml glucose, Life Technologies), 10 ng/ml biotin (Sigma), 0.5% hormone mixture (1 mg/ml apotransferrin, 20 mM putrescine, 4 μM progesterone, 6 μM selenium (formulation based on N2 mix of [Bottenstein & Sato, 1979]) 50 nM hydrocortisone, and 0.5 mg/ml insulin known as DM+, or DM− if lacking insulin (all reagents from Sigma). Each 35 mm Petri dish containing three myelinating culture coverslips was fed three times a week by removing 400 μl of medium and adding 500 μl fresh DM+ for 12 DIV then DM− for the proceeding 12 DIV. Cultures were maintained for 24–35 DIV in an atmosphere of 7% CO_2_ at 37°C and referred to as MC‐Dev. Over time these cultures elaborate myelinated fibers interspaced with organized nodes of Ranvier (Sorensen et al., 2008), with normal periodicity and compaction (Thomson et al., 2008). To assess the effect of mHeps on developmental myelination, cultures were treated at 13 DIV and 20 DIV (1 ng/ml). Furthermore, MC‐Dev cultures underwent a single treatment at 24 DIV to assess if mHeps (1 ng/ml) had any effects on mature myelin. Following which both sets of cultures were fixed and stained at 28 DIV. MC‐Dev cultures were treated with amyloid beta (Aβ)‐peptide (1–42) rat (TOCRIS‐2425) at 16, 19, and 21 DIV (1 μM) or combined with 100 ng/ml mHep7 during feeding. The cultures were fixed and stained at 18, 20, 22, and 24 DIV for myelin quantification (described below). Additionally, conditioned media was collected for CytoTox 96® NonRadioactive Cytotoxicity Assay (Promega G1780).

For generating MC‐Inj, at 24 DIV myelinating cultures were cut using a 11 mm single edge razor blade (WPI, Aston, UK) pressed gently across the center of the coverslip. Details can be found in Boomkamp et al., 2012, 2014. The cut created a focal cell‐free area (650 μm) with a decrease in neurite density and myelination levels adjacent to it and very low numbers of neurites crossing the cut area site, referred to as the lesion. Using these cultures lesion size, neurite density and outgrowth, and myelination can be assessed using immunocytochemistry.

MC‐Inj responds appropriately to treatment with compounds reported to be effective in animal models of CNS injury, corroborating MC‐Inj as a moderate throughput screen for CNS injury (Boomkamp et al., 2012, 2014). The cultures were treated with each mHep at a concentration of 1 ng/ml for a single treatment after 25 DIV and allowed to recover for a further 5 DIV, cultures were then fixed and stained as described below.

### 2.4 Demyelinated cultures (MC‐Demy)

To assess remyelination the myelinating cultures were set up and maintained as described above. At 24 DIV, when many fibers are myelinated, the cultures were demyelinated by overnight incubation with the Z2 antibody (100 ng/ml Hybridoma, a kind gift from Prof C Linington) which recognizes myelin oligodendrocyte glycoprotein (MOG) and rabbit complement (100 μg/ml, Millipore) at 37°C. The demyelinated cultures were then washed with DM− to remove excess complement and treated with the panel of mHeps at 1 ng/ml. At 30 DIV, the cultures were fixed and stained as described below.

### 2.5 Conditioned medium collection

To obtain medium conditioned by the various cultures, MC‐Inj and MC‐Demy were set up. Conditioned media was collected after 25 DIV, for the cut conditioned medium (CCM) and demyelination conditioned media (DCM); this was 24 hr after cultures had been cut and demyelinated respectively. Uninjured culture conditioned medium (UCM) was also collected from myelinating cultures at 25 DIV. CM was also taken after treatment at 26 and 28 DIV (corresponding to days 1 and 3 posttreatment). The CMs were added to MC‐Dev at 16, 19, and 21 DIV. CM was diluted 1:4 with DM− or combined with 1 ng/ml mHep6. At 24 DIV, the cultures were fixed and stained as described below.

### 2.6 Cytokine array screen

Conditioned medium from each type of MC was collected as described above and 1.5 ml was assayed in a Proteome Profiler Rat XL Cytokine Array (R&D Systems, ARY030) using the manufacturer's protocol. The array simultaneously detects levels of 79 rat cytokines, chemokines, growth factors, and other soluble proteins (see https://www.rndsystems.com/products/proteome‐profiler‐rat‐xl‐cytokine‐array_ary0300. Comparisons of CCM and DCM were made to UCM, using semi‐quantitative densitometry analysis on Total Lab Quant Software (TotaLab Limited). Using the circle tool each dot was individually quantified with duplicates averaged and the integrated density values compared.

### 2.7 Oligodendrocyte progenitor cell culture

Oligodendrocyte progenitor cells (OPC) were isolated from cortical astrocyte monolayers generated from postnatal day 1 (P1) SD pups by differential adhesion as previously described (Miron et al., 2013; Noble & Murray, 1984 **)**. OPCs were maintained in serum‐free DMEM‐BS (adapted from Bottenstein & Sato, 1979) containing 0.5 mg/ml insulin in 10 mM HCL (Sigma, UK), glutamine (100 mM, Sigma), human transferrin (0.1 mg/ml, Sigma) and gentamycin (100 mg/ml, Sigma), supplemented with the growth factors; fibroblast growth factor (FGF‐2) at 10 ng/ml and platelet derived growth factor (PDGF) at 2 ng/ml (both Peprotech, UK). The isolated OPCs were plated on poly‐l‐lysine (PLL, 13 μg/ml, Sigma) coated glass coverslips (VWR) in a 24‐well plate at a density of 5,000 cells in 50 μl drop and allowed to attach. They were maintained in DMEM‐BS containing PDGFα and FGF2 for 5 days and then switched to DMEM‐BS lacking growth factors and with or without mHeps at a concentration of 1 ng/ml. Cultures were used for proliferation, morphology, and differentiation assays.

### 2.8 Nanofibres

OPCs were seeded onto PLL coated nanofiber culture inserts (Nanofiber Solutions TM) at a density of 25,000 cells in DMEM‐BS containing PDGFα and FGF2 and incubated for 5 DIV. The OPC medium was then switched to DMEM‐BS containing mHeps at 1 ng/ml for 7 DIV. Purified OPCs sheath length was quantified after mHep treatment by measuring a single internode (PLP+ process) per cell, from cell body to outmost internode and cell size by measuring the surface area of each cell in ImageJ.

### 2.9 Immunocytochemistry

MC were fixed with 4% paraformaldehyde (4% PFA, Sigma) for 20 min at room temperature (RT) and permeabilized with 0.2% Triton X‐100 (Sigma) at RT for 15 min, blocked with PBS with 0.2% porcine gelatin (Sigma) prior to addition of primary antibodies for 1 hr at RT. For extracellular labeling, the cultures were incubated with primary antibodies for 20 min at RT. Oligodendrocyte lineage cells were visualized with the O4 antibody (1:1, mouse IgM hybridoma: Sommer & Schachner, 1981). Cells were washed, incubated with the appropriate secondary Alexa fluorophore‐conjugated antibody (1:500, Life Technologies) for 20 min at RT and fixed in ice‐cold methanol for 15 min at −4°C. For co‐labeling with intracellular antibodies, cells were incubated with primary antibodies, diluted in blocking buffer (containing PBS with 0.1% Triton X‐100 and 0.2% porcine gelatin) for 45 min at RT followed by the appropriate secondary Alexa fluorophore‐conjugated antibodies for 45 min at RT. The cells were washed and mounted in Vectashield (Vector Laboratories, Peterborough, UK).

For intracellular labeling alone, the cultures were fixed in 4% PFA for 20 min at RT followed by washes in PBS and permeabilization in 0.2% Triton‐X100 for 15 min at RT. The primary antibodies were added, diluted in blocking buffer, and the cells incubated for 45 min at RT. Mature myelin (PLP) was visualized using AA3 antibody (1:100, anti‐rat; hybridoma supernatant, Yamamura, Konola, Wekerle, & Lees, 1991), Neurofilament was detected using SMI31 (mouse IgG1, 1:1500, BioLegend). After washing, the cultures were incubated with the appropriate secondary antibodies at RT for 45 min. The cells were washed in PBS followed by dH_2_O, mounted in Vectashield (Vector Laboratories, Peterborough, UK).

### 2.10 EdU cell proliferation assay

For proliferation studies OPCs were maintained for only 2 days in DMEM‐BS containing PDGFα and FGF2. The cells were then switched into DMEM‐BS lacking growth factors for 24 hr (except for the PDGF/FGF condition) before treatment with mHeps, along with immediate incubation with 10 mM of 5‐ethynyl‐2′‐deoxyuridine (EdU) for 18 hr. EdU detection was performed using Click‐iT EdU imaging kit (C10084, Invitrogen) per manufacturer's instructions. The OPCs were then immunolabeled with Olig2 (Rabbit polyclonal, 1:500, Millipore, UK) following which cultures were washed and mounted as previously described.

### 2.11 Microscopy and image analysis

Cells were imaged using Olympus BX51 or LAS AF Leica DM4000 B fluorescence microscopes. For quantitative analysis of injury, neurite density, myelination, and neurite outgrowth, the entire lesion site were imaged adjacent (0–670 μm from the lesion edge) and the actual injury site with each condition being blinded to the experimenter. Similarly, for the MC‐Dev and MC‐Demy quantification of neurite density and myelination, each condition was blinded to the experimenter and standardized random sampling was performed. For neurite density, myelination, and neurite outgrowth studies images were taken at 10× magnification with 20 images per coverslip. Cell counting analysis was made for OPCs and OLs respectively at 10× and 20× magnifications with 10 images per coverslip. The cells of interest were counted per field of view and divided by the total number of DAPI‐positive cells.

### 2.12 Myelination and OPC/oligodendrocyte (OL) quantification

Quantification was carried out using CellProfiler Image Analysis software (Broad Institute) (Carpenter et al., 2006; Lindner et al., 2015). For neurite density, the threshold level pixel value for SMI31 immunoreactivity (IR) was divided by the total number of pixels. The percentage of myelinated axons (PLP) was measured using CellProfiler, which uses pattern recognition software to distinguish between linear myelinated internodes and oligodendrocyte cell bodies. In this manner, we track the co‐expression of myelin sheaths (PLP) and axons (SMI31) and calculate this percentage of myelinated fibers. All experiments were carried out at least three times in duplicate. For OL quantification images adjacent to the lesion were used and OL cells were calculated using a Cellprofiler pipeline which counts the presence of PLP+ cell bodies overlapping DAPI nuclei. OPC/OL cultured on nanofibers were analyzed for cell size and myelin internode length. Both were calculated using ImageJ software. Two measurements per cell were taken from the cell body to the outmost myelin internode 20 cells were used per image selected at random. The cell size was defined by the green (PLP+) pixel threshold compared to the total pixel intensity and individual cells were marked with the region of interest (ROI) tool to allow single cell analysis. All CellProfiler pipelines used in this study are available at https://github.com/muecs/cp.

### 2.13 Neurite outgrowth and lesion size quantification

Ten images of the cut in the MC‐Inj were collected using random sampling. Neurite outgrowth was defined as a SMI31 positive projection which enters and crosses the lesion site. In each image, the number of neurites which cross the lesion site was counted. Any area around the lesion that appeared uninjured was excluded from analysis. The number of neurites per image was averaged across the lesion and termed neurite outgrowth per field of view. Using the same images, the width of the lesion was calculated using ImageJ at 10 fields of view per lesion, averaged and termed lesion width (μm).

### 2.14 HBPs pull down and mass spectroscopy analysis

CCM, DCM, and UCM were affinity purified on an mHep7 column. To make the column a commercial HiTrap NHS‐activated HP column (1 ml, GE Healthcare) was washed extensively with ddH_2_O (20 ml) and 10 mg (1 ml) of mHep7 was introduced onto the column and allowed to react at 15°C for 2 hr. Any unbound mHep7 was washed off the column with ddH_2_O (20 ml) and a small soluble amine (5% ethanolamine in ddH_2_O, Sigma) was added to react with any remaining N‐hydroxysuccinimide (NHS) groups at 15°C for 2 hr. The column was washed with PBS (pH 7.5, 20 ml) and ready for use to capture mHep7 binding proteins. Six milliliters of the different CM was run down the column with a peristaltic pump, following the manufacturer's instructions. Binding buffer and elution buffer was 10 mM sodium phosphate, pH 7 and 10 mM sodium phosphate, 1 M NaCl pH 7, respectively (Both from VWR). The eluted HBPs were desalted and concentrated using Amicon Ultra‐15 centrifugal device (3K, 15 ml; Millipore, UK). The proteins were digested with trypsin, using the FASP protocol (Wiśniewski, Zougman, Nagaraj, & Mann, 2009) and analyzed by LC–MS using an Orbitrap Elite MS (Thermo Scientific) as described previously (Akpunarlieva et al., 2017). Protein identifications were assigned using the Matrix Science MascotDaemon server (Mascot) search engine to interrogate protein sequences in the UniProt database RAT genome, allowing a mass tolerance of 10 ppm for the precursor and 0.6 Da for MS/MS matching.

To obtain quantitative data, UCM, CCM, and DCM were also analyzed by tandem mass tag (TMT) labeling and liquid chromatography–mass spectrometry (LC–MS). The samples were digested with trypsin to generate peptides that were differentially labeled with multiplex tandem mass tags as previously described (Bilić et al., 2018). Samples were then mixed and analyzed by LC–MS. The TMT plates are six‐plex to enable multiplex analysis and the resulting peptides, covalently labeled with TMT tags, were solubilized in 2% acetonitrile with 0.1% trifluroacetic acid and fractionated on a nanoflow uHPLC system (Thermo RSLCnano) before online analysis by electrospray ionization (ESI) mass spectrometry on an Orbitrap Elite mass spectrometer. Peptide separation was performed on a Pepmap C18 reversed phase column (LC Packings). The output from the LC–MS/MS was deconvoluted using ProteomeDiscoverer software, with advice from bioinformaticians at Glasgow Polyomics, and the relative change in abundance of proteins between samples for comparison was determined, with statistical significance assessed by analysis of variance between replicates using Minitab. Any value with a Mascot score of greater than 70 was considered significant and those with Mascot scores below 20 were discounted**.**

### 2.15 Amyloid beta (Aβ) (1–42) and (1–40) ELISAs

MC‐Demy cultures were treated with mHep7 (1 ng/ml) and media collected at 26 and 28 DIV (equating to days 1 and 3 posttreatment, respectively). This CM and mHep7 eluate described above were tested using amyloid beta peptide 1–42 and 1–40 ELISAs (Thermofisher, KHB3441/KHB3481) as per manufacturer's description.

### 2.16 Statistical analysis

GraphPad Prism software was used for data presentation and statistical testing. For simple comparison paired Student's *t* test was employed to determine statistical significance. For multiple condition comparisons, a one‐way repeated measures anova test was employed to data sets followed by Dunnett's multi‐comparison test or Holm–Sidak post hoc correction to calculate potential significant difference. Asterisks are used to represent significantly less than *p < .05, **p < .01, ***p < .001 and inserted onto graphed data. All errors are depicted as standard errors of the mean (SEM). A minimum of three technical repeats/experiment and at least three biological (*n*) repeats were made. Gene Ontology term enrichment analysis was performed in R, using TopGO (Alexa & Rahnenfuhrer, 2016). As a Gene Ontology database, we used the GO term from UniProt. To correct for multi‐testing, we multiply the *p* value by the amount of statistical test that was performed.

## 3 RESULTS

### 3.1 Selectively desulfated mHeps promote neurite outgrowth and myelination in myelinating culture‐injured (MC‐Inj)

**Figure 1:**
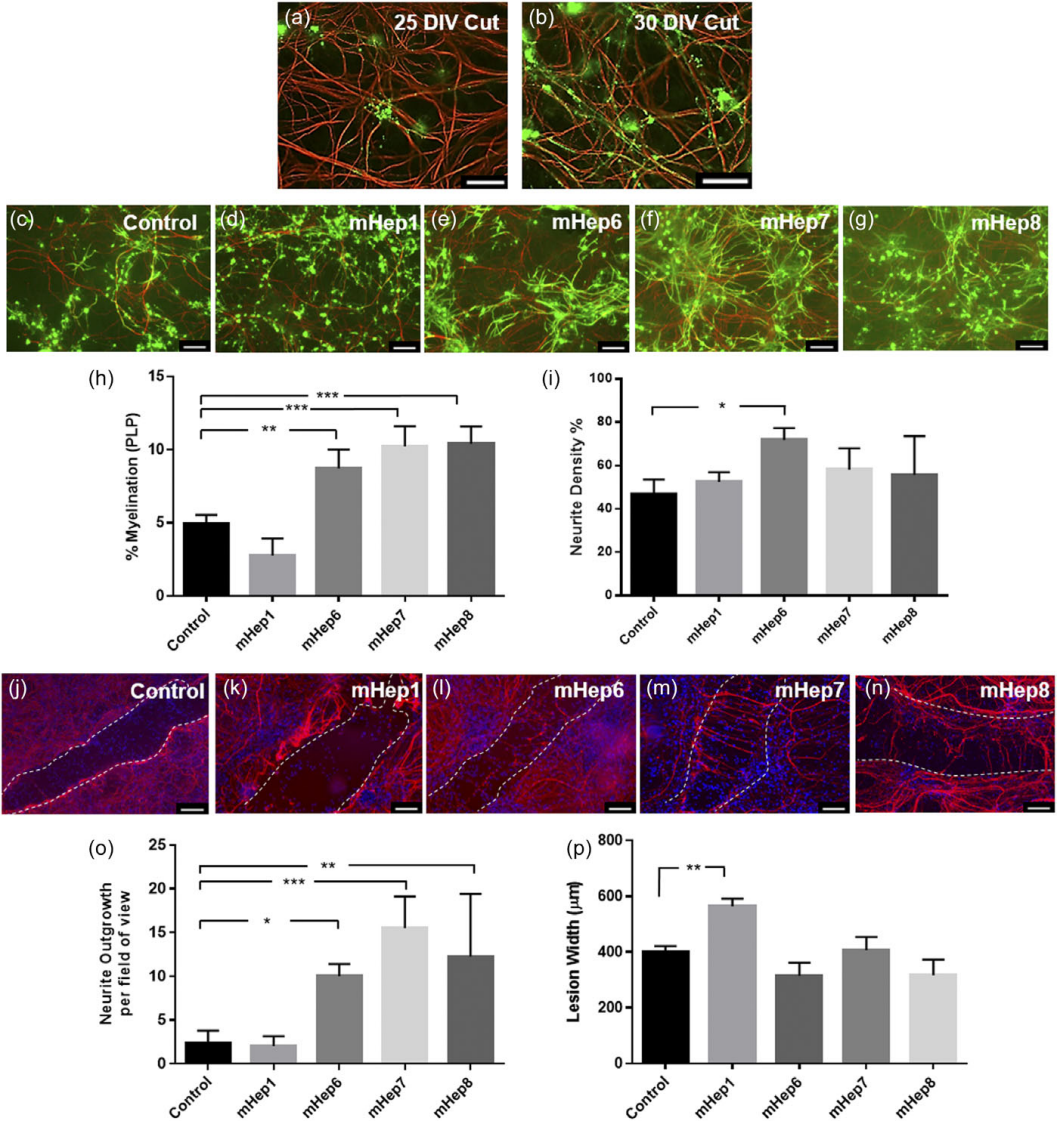
LS‐mHep promote myelination and neurite outgrowth (a,b) High power images of demyelination adjacent to the lesion after injury and the subsequent myelination occurring after 5 days. Representative images of MC‐Inj of a control cut (c) and after a single treatment with LS‐mHep6‐8 (e–g) showing a promotion of myelination adjacent to the lesion which is adjacent and just out of view in the images. In contrast, the HS‐mHep1 showed a nonsignificant decrease in the levels of myelination (d). Quantification of the images are shown in h (*p* = .0017, *p* < .0001 and *p* = .0001 for mHep6, 7 and 8 respectively). Only mHep6 significantly promoted neurite density adjacent to the lesion when compared to control cultures (*p* = .0127) (i). Representative images of the lesion after cutting with a scalpel blade in MC‐Inj (j), and after HS‐mHep1 treatment showing no effect on neurite outgrowth (k). Significant neurite outgrowth across the lesion was seen after treatment with LS‐mHep6‐8 compared to control untreated cultures (*p* = .0172, *p* < .0001 and *p* = .0029 for mHep6, 7, and 8, respectively) (l–o). Representative image of lesion size is shown in (j–n) identified by dashed line. An increase in lesion width following treatment with HS‐mHep1 (564.5 μm) was seen, compared to the control lesion (400 μm *p* = .0026). LS‐mHep6‐8 had no effect on lesion width (p). Statistical test used was one‐way anova with post hoc Dunnett's multi‐comparison correction. Scale bar, 25 μm and 50 μm, error bars SEM, SMI31‐red, PLP‐green (*n* = 6; technical replicates = 3)

The effects of a number of selectively desulfated mHeps (Higginson et al., 2012; O'Neill et al., 2017) on neurite density, myelination, neurite outgrowth, and lesion size following injury were assessed using the MC‐Inj model. High power images of demyelination after injury and the subsequent myelination after 5 days can be seen in Figure 1a,b. Figure 1c–g shows representative images of myelination adjacent to the lesion in cut control (c) and mHep treated (d–g) MC‐Inj. It can be seen that the percentage of myelinated fibers was significantly higher after treatment with the low‐ or de‐sulfated mHeps6‐8 increasing to 8–10% from 5% in untreated control (*p* = .0017, <.0001, and .0001 for mHep6, 7, and 8, respectively), but treatment with HS‐mHep1 resulted in a decreasing trend to 2.5% although this appears nonsignificant (Figure 1d,g, quantification in panel h). Neurite density was analyzed adjacent to the injury site and a significant increase (*p* = .0127) was observed after treatment with LS‐mHep6 compared to the untreated control, whereas the other mHeps1, 7, and 8 had no significant effect on neurite density (Figure 1i). Figure 1j–n show representative images of neurites crossing the lesion and the lesion size in control and treated cultures. Quantification of the average number of neurites crossing the lesion is shown in Figure 1o. The LS‐mHeps 6–8, all significantly promoted neurite outgrowth across the lesion compared to the untreated control (*p* = .0172, <.0001, and .0029 for mHep6, 7, and 8, respectively). The HS‐mHep1 did not promote neurite outgrowth after treatment, with neurite numbers being similar to the untreated control (~3 neurites/field of view). Finally, the width of the lesion was quantified after mHep treatment (Figure 1p). HS‐mHep1 treatment resulted in a significant increase in lesion size (564 ± 27.5 μm, *p* = .0026) compared to the untreated control (400 ± 20.0 μm, Figure 1j,k, and p). In contrast, the LS‐mHeps6–8, did not significantly affect the lesion size, with average lesion sizes being, 315.2 ± 46.8 μm (mHep6), 405 ± 48.6 μm (mHep7), and 317 ± 56.2 μm (mHep8).

### 3.2 LS‐mHeps promote oligodendrocyte process extension

**Figure 2:**
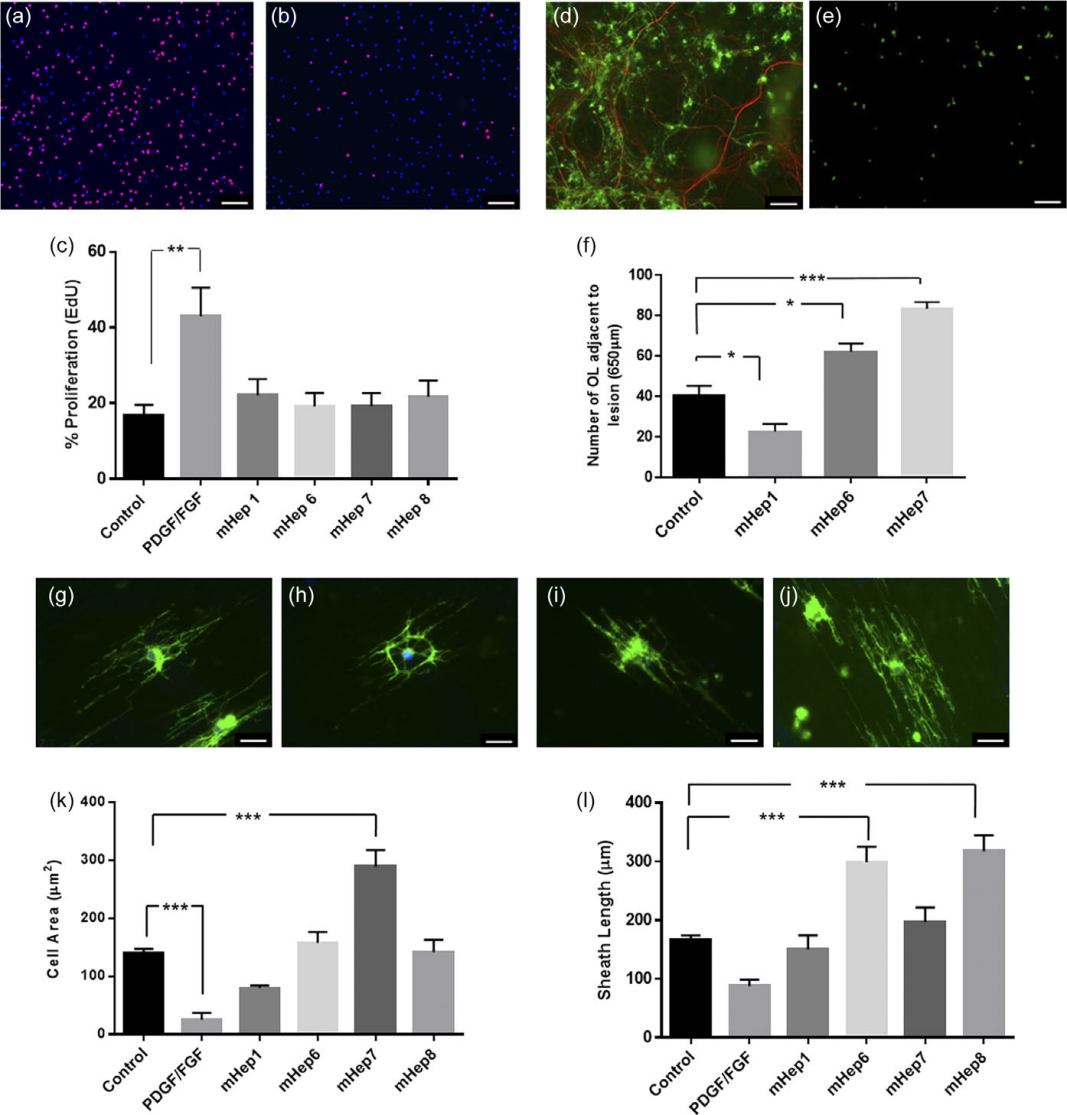
LS‐mHeps do not affect OPC proliferation but promote the number of OL adjacent to the lesion and process extension on nanofibers. (a–c) mHep treatment had no effect on the proliferation of OPCs. a and b are representative images fluorescently labeled with DAPI‐blue and EdU‐red of PDGF/FGF treated and control OPCs, respectively. Quantification of proliferation was determined by percentage of EdU positive cells which demonstrated no difference between control and mHep treated OPCs (c). (d–f) LS‐mHep6 and 7 treatment caused an increase in the number of OLs adjacent to the lesion (*p* = .0133 and *p* = .0002, respectively). (d) Microscope image stained for PLP (green) and SMI‐31 (red) at lesion edge of MC‐Inj. (e) Same image quantified for PLP‐IR (OL) after LS‐mHep treatment. In contrast, HS‐mHep1 treated cultures showed a significant decrease in the number of OLs compared to control cultures (*p* = .0336). Representative images of PLP stained OPCs plated on nanofibers in the presence of DMEM‐BS (g), HS‐mHep1 (h), LS‐mHep6 (i), and LS‐mHep7 (j). (k) Quantification of cell area demonstrated that LS‐mHep7 treatment resulted in an increase in the OL cell size, compared to the untreated control (*p* < .0001). (l) Quantification of myelin sheath length demonstrated that LS‐mHep6 and 8 treatments statistically promoted sheath length compared to controls (*p* = .0001 for both). As expected GF treated OPCs had a reduced OL cell size (*p* = .0005) with limited process extension along the nanofibers (k, l). Statistical test used was one‐way ANOVA with post hoc Dunnett's multi‐comparison correction. Scale bars, 100 μm and 25 μm, error bars SEM (*n* = 4; technical replicates = 3)

To determine the effect of the mHeps directly on OPCs, we treated purified OPCs with 1 ng/ml of mHeps. First, we established that OPC proliferation was unaffected by any mHep treatment when compared to DMEM‐BS control, and PDGF/FGF2 treatment, a growth factor cocktail known to promote OPC proliferation (*p* = .0032; Figure 2a–c). Representative images of the control and PDGF/FGF treated OPCs are shown in Figure 2a,b, respectively. HS‐mHep1 treatment induced a significant decrease in OL numbers adjacent to the lesion compared to the untreated control (Figure 2d–f; *p* = .0336). Conversely, LS‐mHep6 and 7 treatment resulted in a significant increase in the number of OLs adjacent to the lesion site compared to control (Figure 2d–f; *p* = .0133 and .0002).

To investigate whether mHeps directly affect OPC process wrapping we used nanofibers which allow the study of ensheathment in the absence of dynamic neuronal signaling (Lee et al., 2012). The OL sheath length in DMEM‐BS alone (control) averaged 166.6 ± 20.4 μm with the growth factor control (PDGF/FGF) cell size only reaching 87.3 ± 31.2 μm (Figure 2g–l). HS‐mHep1 had no significant effect on the sheath length with the average length reaching 150.6 ± 57.9 μm (Figure 2h–l). Similarly, the LS‐mHep7 had no significant effect on the sheath length with average lengths measuring 196.9 ± 60.2 μm (Figure 2j–l). The cell area of OLs was significantly increased following treatment with LS‐mHep7 (289.2 ± 49.8 μm^2^, *p* < .0001) compared to control (139 ± 16.9 μm^2^; Figure 2j,k). Treatment with LS‐mHep6 and 8 did not affect cell area (157.9 ± 41.9 and 141.9 ± 36.9 μm^2^, respectively), however, HS‐mHep1 treatment caused a nonsignificant decrease in the overall cell area (79.7 ± 8.3 μm^2^; Figure 2h), Moreover, LS‐mHep6 and 8 treatment induced a significant increase in the OL sheath length compared to control with an average length of 298.6 ± 84.3 μm and 317.4 ± 66.7 μm, respectively (Figure 2i–l; *p* = .0001 for both).

### 3.3 mHep treatments have no effect on myelination levels in MC‐Dev

**Figure 3:**
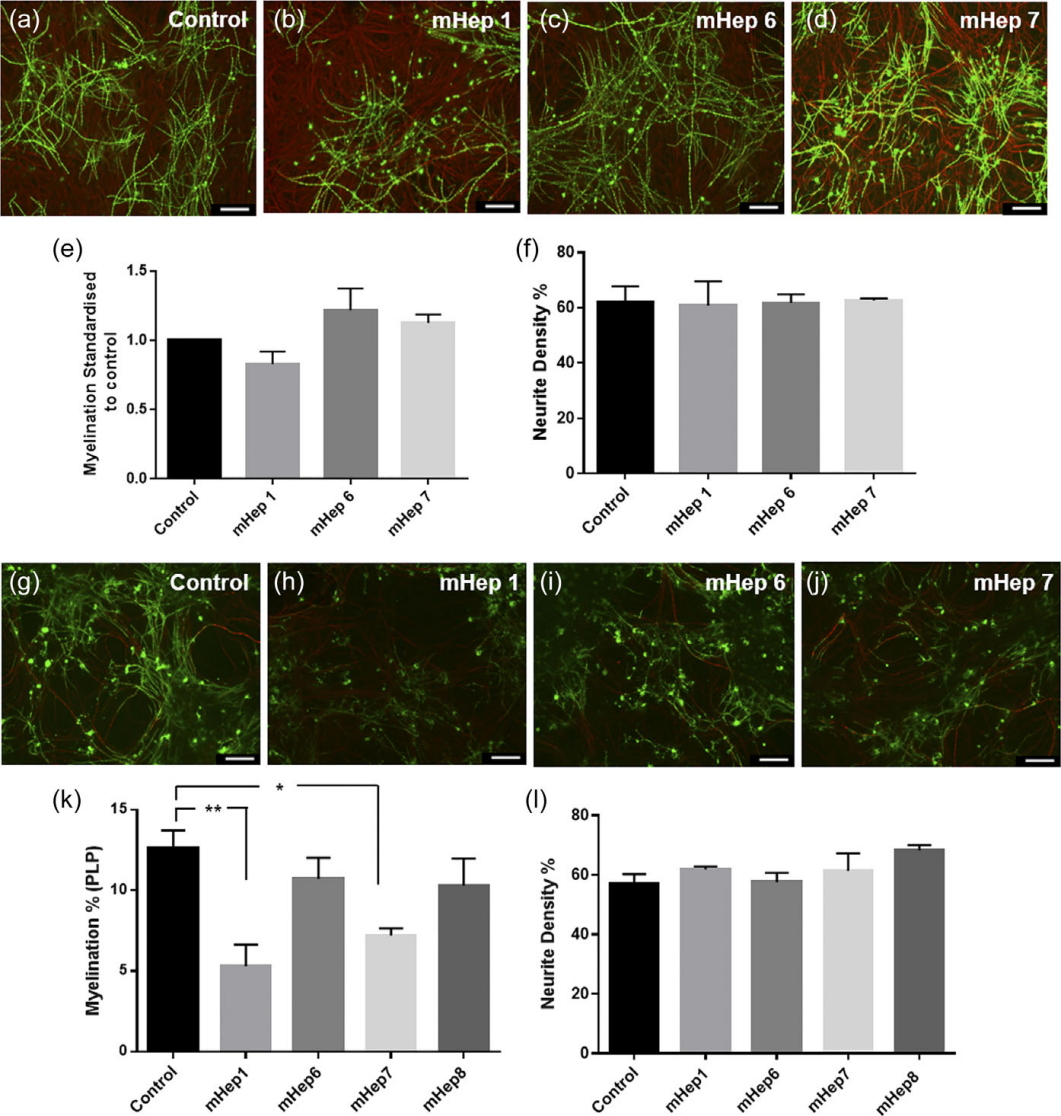
2‐*O*‐sulfated mHeps had no effect on the development of myelination but mHep1 and the *N*‐sulfated isoform affected myelinated fibers. (a–d) Representative images of MC‐Dev control or mHep (1 ng/ml added at 13 and 20 DIV) treated cultures showing myelinated fibers, SMI31‐red and PLP‐green. (e) Quantification of myelination relative to the untreated control showing no significant difference in myelination levels after mHep treatment. (f) Neurite density was also unaltered by mHep treatment. (g–j) Representative images of MC‐Dev control or mHep (1 ng/ml added at 24 DIV) treated cultures. Quantification of myelination (k) demonstrated a reduction in myelination levels post‐HS‐mHep1 (*p* = .0033) and LS‐mHep7 (*p* = .0163) treatments on the mature cultures. While neurite density remained unchanged (l). Scale bars 100 μm, error bars SEM (*n* = 4; technical replicates = 3)

Rat myelinating cultures (MC‐Dev) were treated with mHeps1, 6, and 7 (1 ng/ml) after 13 DIV or 24 DIV, fixed and stained at 28 DIV and the percentage of myelination and neurite density within the cultures quantified. Figure 3a–d illustrates representative images of control and treated cultures after mHeps were added from 13 DIV while Figure 3g–j show their effects when added from 24 DIV. Myelination and neurite density were quantified and shown in Figure 3e–k and f–l, respectively. It can be seen that mHep treatments did not significantly affect either myelination levels or neurite density in MC‐Dev when added from 13 DIV compared to untreated control. In contrast, when treatment occurred at 24 DIV, once more fibers were myelinated, it was seen that the addition of mHep1 and mHep7 resulted in a significant decrease in myelination when compared to untreated controls (Figure 3k; *p* = .0033 and .0163, respectively), although neurite density was not affected (Figure 3l).

### 3.4 LS‐mHep treatment enhances CNS remyelination

**Figure 4:**
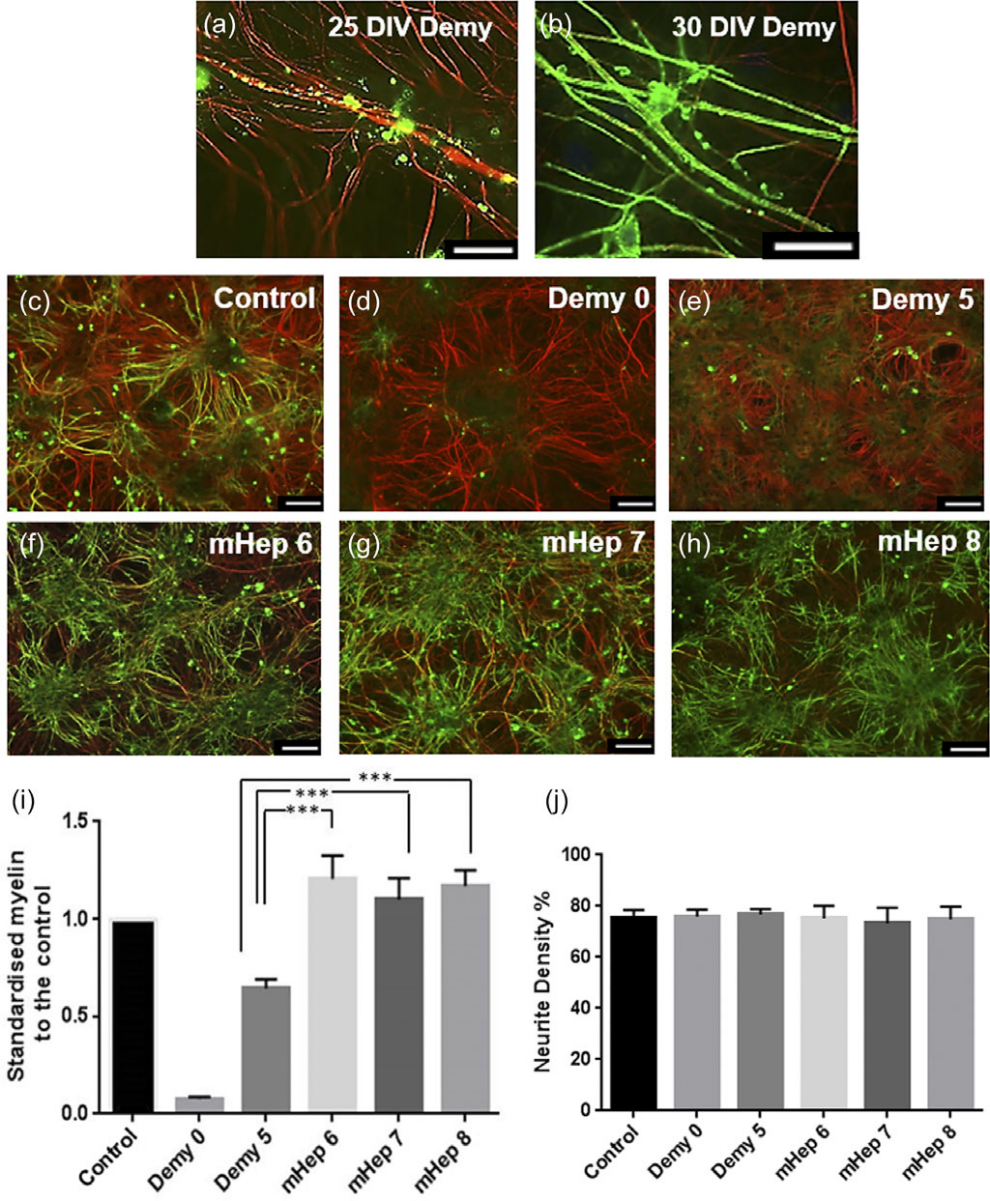
LS‐mHeps have significant effects on remyelination in MC‐Demy. Myelinating cultures were allowed to mature until 24 DIV at which point they were demyelinated via overnight incubation with the anti‐MOG specific antibody Z2 (100 ng/ml) and rabbit complement (100 μg/ml). Treatment occurred at 25 DIV (1 ng/ml) and then cultures were allowed to recover till 30 DIV, at which point they were immunolabeled with SMI31 (red, neurites) and AA3 (green, myelin). (a,b) High power images showing the loss of myelin immediately after demyelination (Demy0) and the remyelination after 5 days. (c–h) Representative images of MC‐Demy treated with anti‐MOG and complement on 25 DIV (d) and 30 DIV (e) and treated with the mHeps (f–h). (i) Quantification of myelination showing treatment with mHeps resulted in an 87, 71, and 81% increase in remyelination compared to the nontreated demyelinated control culture on day 5 (Demy5) for LS‐mHeps6, 7, and 8 respectively (one‐way ANOVA with Holm–Sidak multiple comparison, *p* = .0002, .0004, and .0002). (j) Quantification of neurite density showed no change in density with treatment implying no adverse toxic effects on the cultures. Scale bars 25 and 100 μm, error bars *SEM* (*n* = 4; technical replicates = 3)

Because the developmental myelination in MC‐Dev was essentially unaffected by treatment with mHeps, we hypothesized that the injury environment produced by MC‐Inj may be essential to their effectiveness in promoting myelination. We propose that similar effects would be seen in a different CNS injury environment. To address this, we used MC‐Demy, in which MC‐Dev cultures were demyelinated with complement and anti‐MOG antibody followed by a single treatment with mHeps (1 ng/ml), and maintained until 30 DIV, followed by staining for PLP and SMI31 to visualize myelination and neurites respectively. Figure 4a,b shows high power images of the loss of myelin sheaths immediately after demyelination (Demy0) and the subsequent remyelination after 5 days. Figure 4c–h shows representative images and Figure 4i,j shows the quantification of myelination and neurite density respectively. It can be seen that LS‐mHeps treatment increased the percentage of myelinated fibers by 70–80% compared to a nontreated demyelinated culture on day 5 referred to as Demy5, without affecting neurite density (*p* = .0002, .0004, and .0002 for mHep6, 7, and 8, respectively). This suggests that the LS‐mHeps enhance remyelination in this injury environment, absent of any toxic effects on neural cells.

### 3.5 Conditioned medium collected from MC‐Inj (CCM) reduces CNS myelination

**Figure 5:**
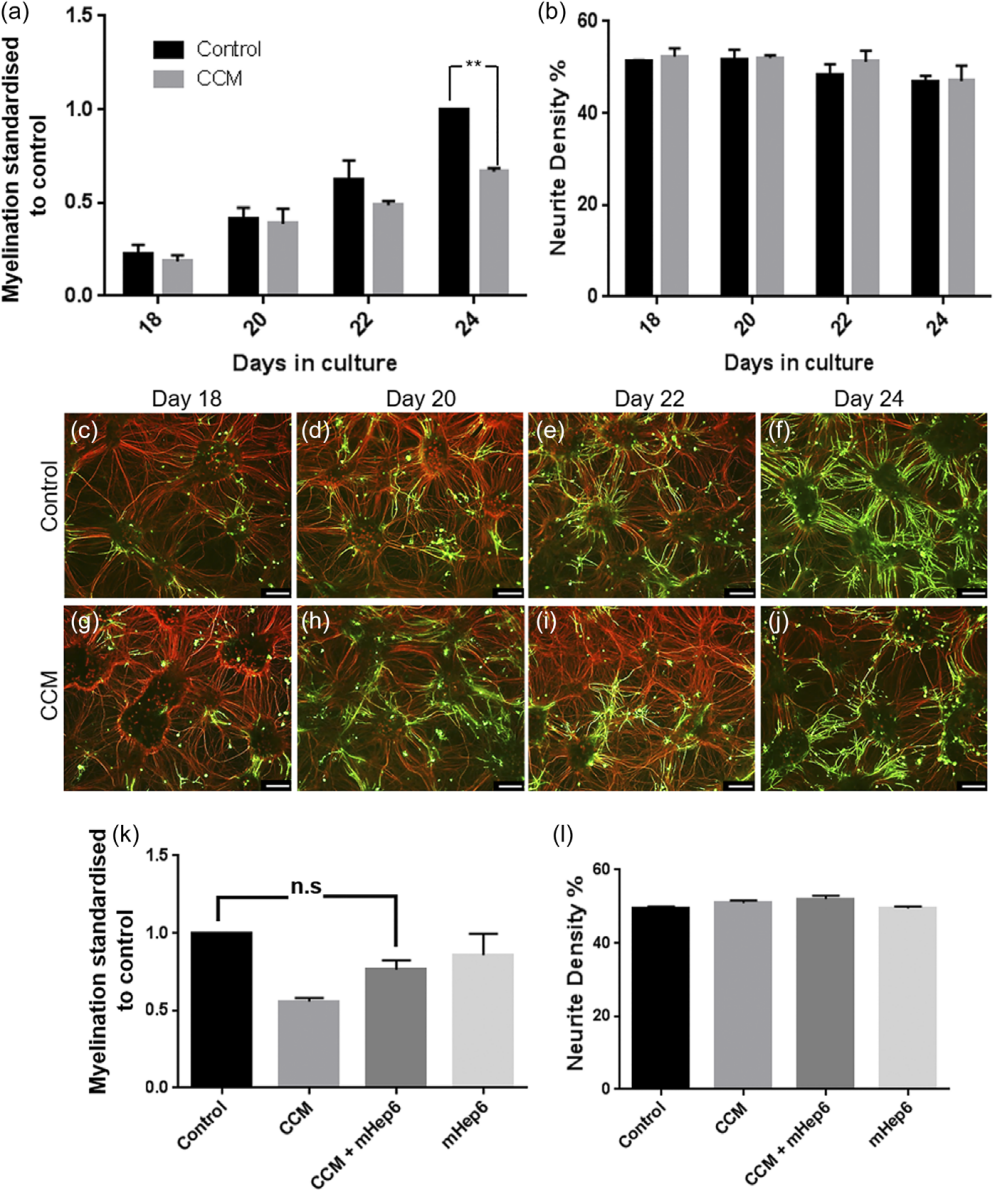
Cut conditioned medium (CCM) significantly reduces developmental myelination, which is partially overcome by LS‐mHeps. CCM was collected from MC‐Inj at 25 DIV and used to treat MC‐Dev cultures at 16, 19, and 21 DIV (1 in 4 dilution). (c–j) Representative images of control and CCM treated cultures, immunolabeled with SMI31‐red and PLP‐green. (a,b) Quantification of myelination and neurite density over time after CCM treatment showing no difference in the development of myelination between CCM treated and control cultures at 18, 20, and 22 DIV. However, at 24 DIV, there was a significant 33% reduction in myelination of the CCM treated cultures compared to the control (*p* = .0031). (k,l) Quantification of myelination and neurite density of MC‐Dev after a combined treatment of CCM and LS‐mHep6 showed an increase in the number of myelinated fibers so as to be nonstatistically different from the untreated control. Quantification of neurite density after treatment with CCM showed no adverse effects on neurites (one‐way anova with Dunnett multiple comparison). Scale bars 100 μm, error bars *SEM* (*n* = 3; technical replicates = 3)

Since we only observed effects on myelination using LS‐mHeps in MC‐Dev after a cut or antibody‐mediated demyelination, we hypothesized that mHeps exert their effects by modulating factors released by injury. To address this, we collected conditioned medium from MC‐Inj (CCM; 1 in 4 dilution) and added it to MC‐Dev at 16, 19, and 21 DIV, fixing and staining the cultures at 18, 20, 22, and 24 DIV with anti‐PLP and the SMI31 antibody. Treatment with CCM resulted in a 33% reduction in myelination compared to control at 24 DIV (Figure 5a) suggesting the lesion in MC‐Inj releases factors which are inhibitory to CNS myelination. This is unlikely to be nonspecific toxicity as neurite density remains unaffected (Figure 5b). Co‐treatment with CCM and LS‐mHep6 indicated a trend towards the rescue of CCM‐induced hypomyelination (assessed by the level of myelination at 24DIV), though this difference did not reach statistical significance (*p* = .1163). These data suggest that the LS‐mHep6 may modulate the properties of proposed heparin binding factors that are induced by injury (Figure 5k,l). Representative images are seen in Figure 5c–j.

### 3.6 Secreted chemokine/cytokine profile is altered after MC‐Inj and MC‐Demy

**Figure 6:**
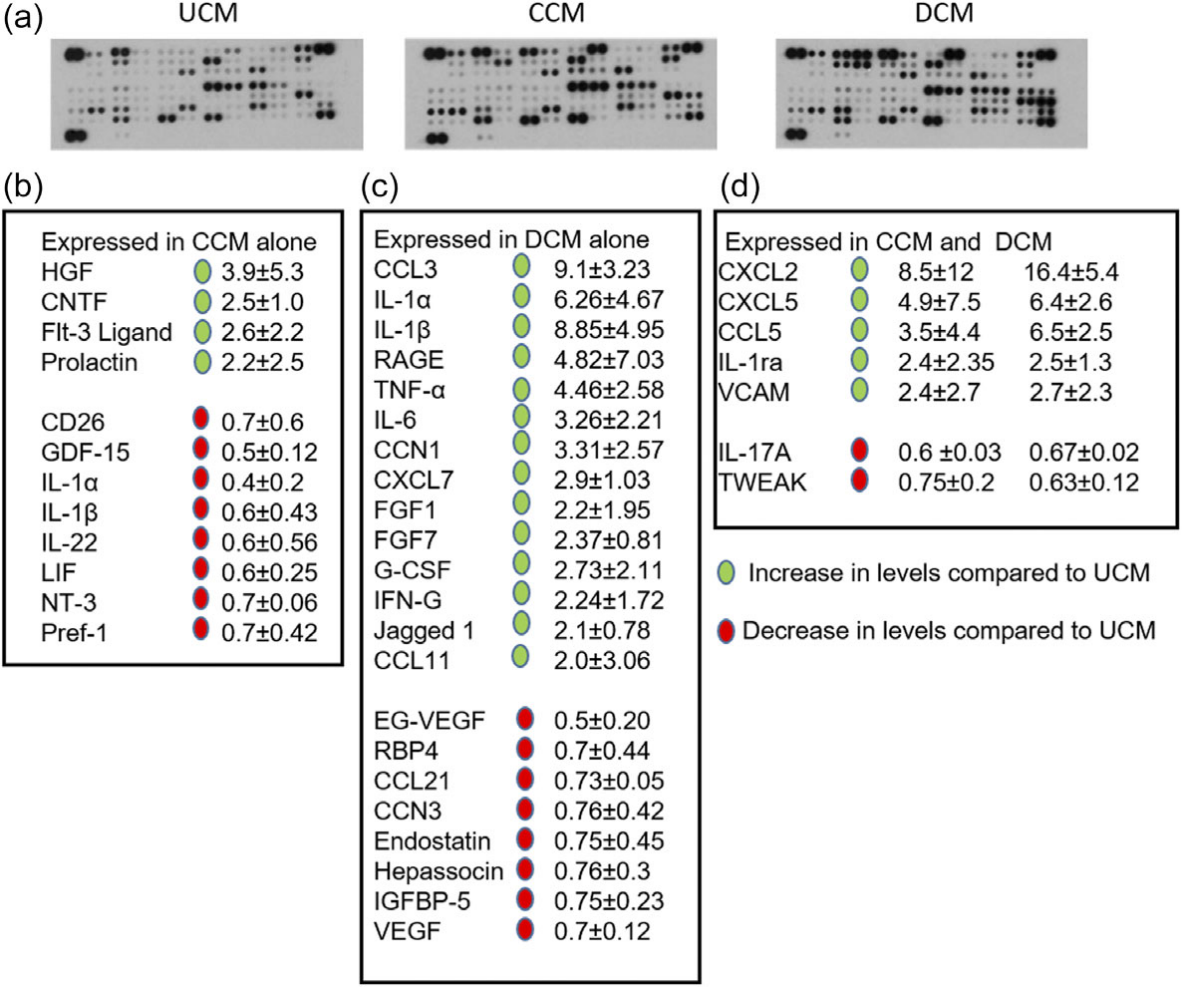
Differences in chemokine/cytokine secretion profile after CNS injury. Cut conditioned media (CCM), demyelinated conditioned medium (DCM), and control conditioned medium (UCM) were collected from MC‐Inj, MC‐Demy, and MC‐Dev, respectively, on 25 DIV and the cytokine profile assessed using a rat cytokine array. (a) Dot blots of the cytokine arrays for each of the conditioned media. (b–d) Tables illustrating the fold changes with (±*SD*) of expression changes in both CCM (b), DCM (c), and CCM/DCM (d) compared to UCM, the green and red spheres indicate >2‐fold increase and <0.8‐fold decrease respectively (*n* = 3; technical replicates = 2)

As a first step to identifying molecules involved in the effects of mHeps described above, we took a candidate approach, reasoning that chemokines and cytokines which bind heparin/HS could be implicated. To identify specific chemokines and cytokines released in CCM and DCM, we conducted a protein array of conditioned media (see methods for details of array). Figure 6b–d illustrates the changes in the secreted cytokines standardized to the UCM. Green and red circles indicate only these factors that had a >2‐fold increase or < 0.8‐fold decrease, respectively. Several factors were upregulated in both CCM and DCM including CXCL2, CXCL5, and CCL5 (average fold increase of 8.5/16.4, 4.9/6.4, and 3.5/6.6 for CCM and DCM, respectively). Striking differences in secreted cytokine profiles were also seen between the two injuries. Many immune‐associated factors including CCL3, IL‐1α, IL‐1β, IL‐6, and TNF‐α were increased in DCM by an average fold change of 9.1, 6.3, 8.9, 3.3, and 4.5, respectively. Interestingly, the same proteins showed no change in CCM, apart from IL‐1α and IL‐1β, for which a decrease was observed (0.4 and 0.6, respectively). The proteins which were exclusively increased in CCM were trophic factors including HGF, CNTF, Flt‐3 ligand, and prolactin, which displayed corresponding average fold changes of 3.9, 2.5, 2.6, and 2.2. Comparing the data set as a whole we observed a significant difference in the proteome profiler between the UCM and the CCM/DCM (*p* = .0008 and .0004, respectively), suggesting a clear shift in the secretome postinjury. Additionally, there was a significant difference between the CCM and DCM identified in this array (*p* = .0047), suggesting some level of injury‐type specificity in the CNS injury secretome.

### 3.7 Mass spectrometry analysis of conditioned medium from MC‐Inj and MC‐Demy

**Figure 7:**
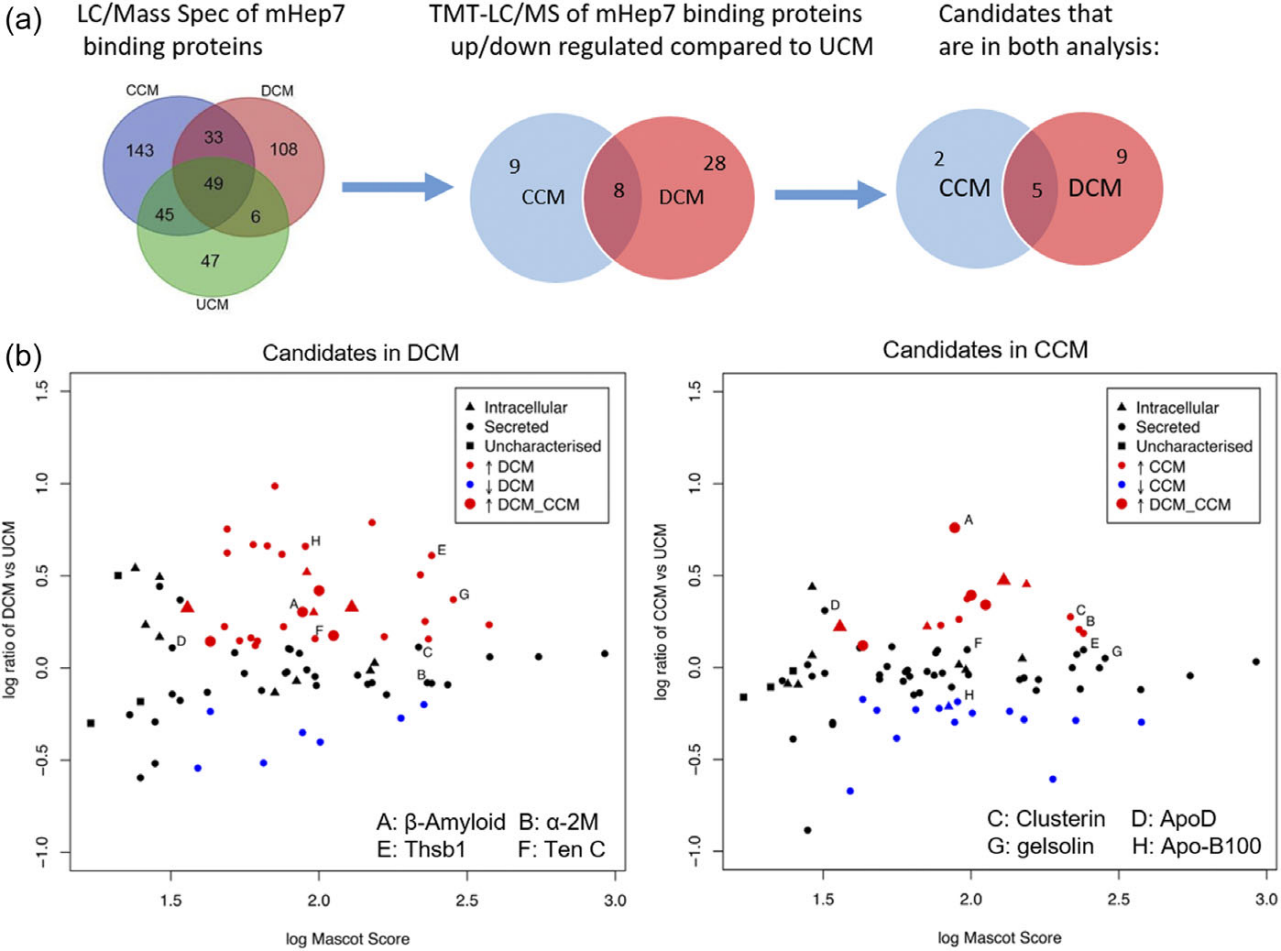
Modified heparin 7 pulldown experiment of CM from MC‐Inj (CCM) and MC‐Demy (DCM). Condition media collected from control cultures (UCM), MC‐Inj (CCM) and MC‐Demy (DCM), was run through a mHep7 column, to select mHep7 binding proteins and identify potential candidates (6 ml CM used per each pulldown, CM a combination of at least *n* = 5). (a) Venn diagram depicting the number of candidates both unique to and shared by each CM. It can be seen that the number of candidates obtained decreased as methods were modified from a nonquantitative LC/MS to TMT‐LC/MS. (b) Graph generated using R package UpsetR, a matrix‐based layout showing the intersections of all three CM data sets. Large symbols indicate the fold increase in shared candidates in DCM and CCM while small red symbols are candidates increased when compared to control and blue symbols are candidates decreased when compared to control. Circles represent secreted candidates, with triangles depicting intracellular candidates and squares for candidates that have no reports in the literature a–h are interesting candidates based on literature searches identified for discussion

As an unbiased approach to identify molecules involved in the effects of mHeps, we used affinity proteomics to explore directly bound proteins that might be mediating the biological responses. To assess which proteins present in the UCM, CCM, and DCM interact with LS‐mHep7, we performed a protein pull‐down using an mHep7 affinity column, followed by mass spectroscopy (MS) analysis on the eluted samples. Number and distribution of specific and shared proteins can be seen in the Venn Diagram (Figure 7a). Overall 431 proteins were identified. CCM contained 143 unique proteins; DCM contained 108. About 33 proteins were shared between CCM and DCM whereas 47 were specific to UCM. To validate the overall data, we performed gene ontology analysis and initially determined the GO enrichment *E*‐value for the entire data set including all three intersections. HBPs (GO:0008201) was the highest enriched term with an *E*‐value of 5.4 e−15, validating the methodology for pulling down proteins related to heparin binding. Due to the nonquantitative nature, and therefore the inability to make direct comparisons between samples, we carried out TMT LC–MS analysis of the conditioned media.

### 3.8 TMT–LC/MS analysis of conditioned medium from MC‐Inj and MC‐Demy

TMT labeling allows the comparison of peptide levels between multiple samples in a single LC–MS run. This multiplexing circumvents problems with reproducibility in serial LC–MS analyses and allows relative quantitation in comparison to control samples. We were able to compare the abundances of LS‐mHep7 binding proteins present in each of the CM. Protein levels in the injury CM were standardized to UCM allowing us to state a fold change (FC) after injury. Figure 7b shows LS‐mHep7 binding proteins with a fold change of >1.3 (red) or <0.8 (blue) after injury and a Mascot score of >29 (higher Mascot scores indicate increased confidence in the protein hits). There were 8 mHep7 binding proteins that had elevated abundances after both MC‐Inj and MC‐Demy represented in the plots by large red symbols (Figure 7b). These include growth hormone releasing hormone receptor which had a massive increase of 327.00 and 136.50 for CCM and DCM, respectively (although with a weak Mascot score of 30). Additionally, amyloid beta A4 protein (APP) increased 5.7‐ and 2.0‐fold in CCM and DCM (Table 2c). There were nine proteins which demonstrated an elevated abundance uniquely in the CCM (Table 2a) including apolipoprotein D, clusterin, calsyntenin‐3, and alpha‐2‐macroglobulin which showed corresponding fold changes of 2.0, 1.9, 1.8, and 1.6 compared to UCM. There were 23 proteins which demonstrated elevated levels uniquely in the DCM after binding to mHep7 (Table 2b). These proteins are diverse in both structure and function from the large lipid transporter apolipoprotein B‐100 (4.57 FC), ECM glycoproteins thrombospondin (4.07 FC) and tenascin C (1.44 FC), protease inhibitor alpha‐1‐macroglobulin (4.21 FC), and actin‐binding protein gelsolin (2.35 FC).

**Table 2:** TMT‐LC/MS identified candidates: List of complete candidates that were identified with Mascot scores greater than 30 and fold increase greater than 1.3 in (a) CCM only, (b) DCM only, and (c) both CCM and DCM

(a) CCM only fold increase		
Protein	CCM FC	Mascot score
Galectin‐3‐binding protein	2.36	97
**Apolipoprotein D**	**2.04**	**32**
**Clusterin**	**1.88**	**217**
Calsyntenin‐3	1.83	91
Carboxylic ester hydrolase	1.7	79
Plakoglobin	1.67	71
**Alpha‐2‐macroglobulin**	**1.61**	**232**
Complement C2	1.53	240

(b) DCM only fold increase		
Protein	DCM FC	Mascot score
Antithrombin	6.14	151
Inter‐alpha‐trypsin inhibitor	5.66	49
Complement component 4A	4.66	60
Ac2‐120	4.6	67
**Apolipoprotein B‐100**	**4.57**	**90**
Alpha‐1‐macroglobulin	4.21	49
Igh‐6 protein	4.13	75
**Thrombospondin 1**	**4.07**	**240**
Inter‐alpha trypsin inhibitor	3.2	220
Complement C5	2.77	29
**Gelsolin**	**2.35**	**284**
Liprin‐alpha‐3	2.34	34
Plasminogen activator inh 1	1.79	228
Alpha‐2 antiplasmin	1.72	375
Complement C1q subunit C	1.68	48
Glia‐derived nexin	1.67	76
Aa1018	1.48	166
Periostin	1.45	59
**Tenascin C**	**1.44**	**97**
Complement C3	1.43	234
Heat shock cognate 71 kDa	1.4	54
Spondin‐1	1.4	62
Noelin	1.32	61

(c) CCM and DCM fold increase			
Protein	CCM FC	DCM FC	Mascot score
Growth horm releasing horm R	327	136.5	30
**Amyloid beta A4 protein**	**5.76**	**2.01**	**88**
Tubulin alpha‐1A chain	2.98	2.13	129
Aplp1 protein	2.75	1.47	29
Fruct‐bisphosphate aldolase A	2.48	2.63	100
Complement inhibitory factor H	1.32	1.4	43
Xylosyltransferase 1	1.66	2.11	36

Candidates that are secreted and of interest in these cultures are highlighted in bold and reflect those indicated in Figure 7b

### 3.9 A focus on amyloid beta (Aβ) 1–42 which is present in the DCM‐mHep7 eluate

**Figure 8:**
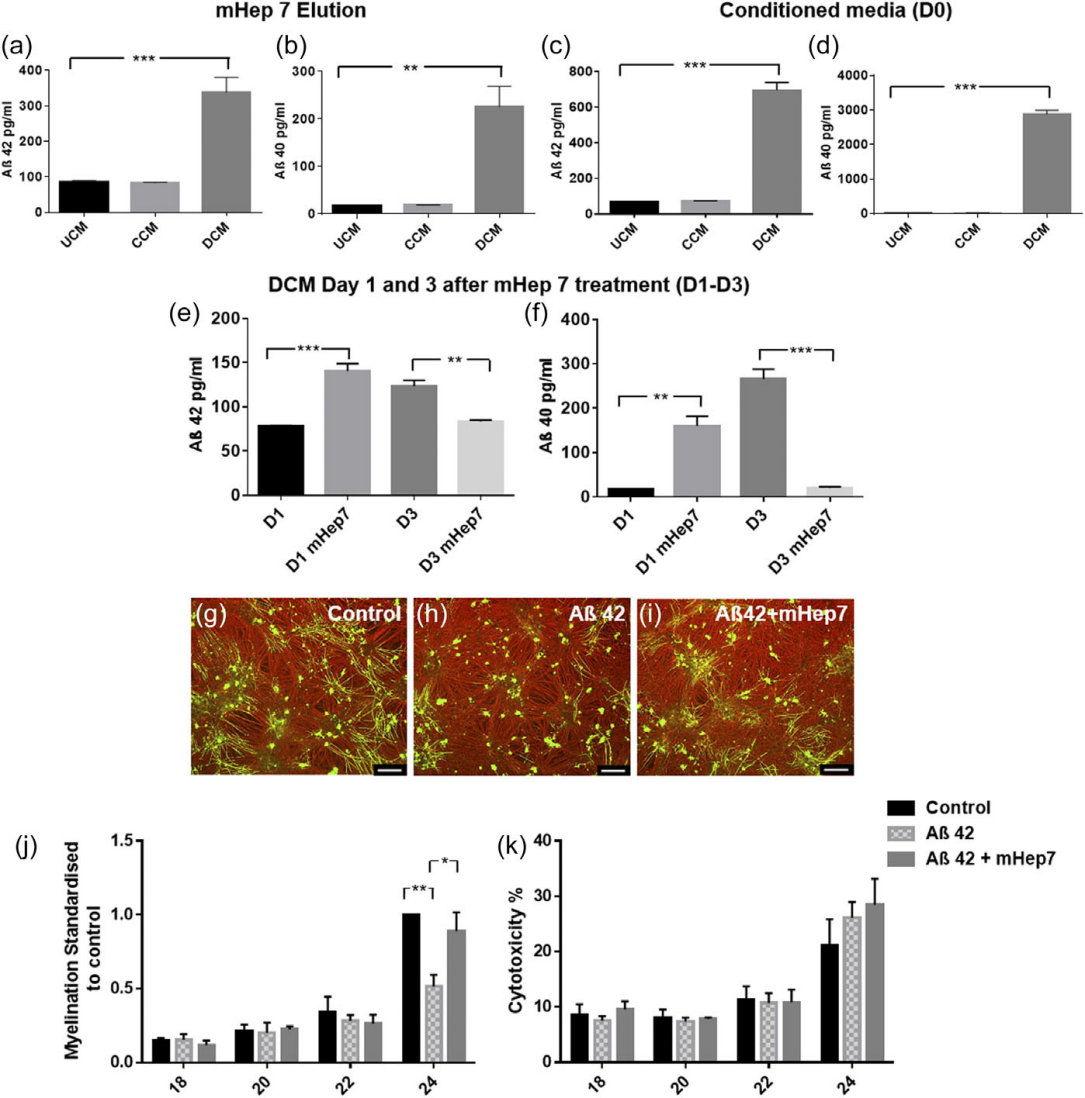
Validation of amyloid beta as a candidate for demyelination. (a,b) ELISAs of the mHep7 eluate. Concentration of Aβ 1–42 (*p* = .0007) and Aβ 1–40 (*p* = .0017) is significantly increased in the DCM mHep7‐eluate compared to the UCM mHep7 eluate. (c,d) ELISAs of CM after injury (day 0). There was a large increase in the level of Aβ 1–42 and Aβ 1–40 peptide secretion immediately after demyelination (D0) compared to the uninjured control (both *p* < .0001). (e,f) MC‐Demy cultures were treated with mHep7 following demyelination. CM was collected at 26 and 28 DIV (corresponding to days 1 and 3 posttreatment). (e) Graph shows a statistically significant increase in Aβ 1–42 present in DCM 24 hr after mHep7 treatment (D1) compared to untreated control (*p* = .0003). However, by day 3 posttreatment (D3), there was a significant reduction in Aβ 1–42 present in the CM following mHep7 treatment (*p* = .0096). The same pattern was observed with Aβ 1–40 with treatment leading to an increase in concentration shortly after treatment at D1 (*p* = .0010), but by D3 there seems to be a significant decrease in the level of Aβ 1–40 following mHep treatment compared to untreated control (*p* = .0001). (g–k) Cultures were treated with Aβ 1–42 (1 μM) at 16, 19, and 21 DIV (co‐treatments with mHep7 at 100 ng/ml). (g–i) Representative images of control, Aβ treated and Aβ + mHep7 treated cultures at 24 DIV, immunolabeled with SMI31‐red and PLP‐green. (j) Quantification of myelination over time showing a significant decrease in developmental myelination at 24 DIV after Aβ treatment (*p* = .0082) this is overcome by mHep7 co‐treatment (*p* = .0321). Quantification of neurite density over time suggests no effect of either treatment on neurite density (data not shown). (k) LDH release cytotoxicity assay demonstrating no statistically significant effect on cell death at 24 DIV following Aβ treatment or mHep7 co‐treatment. Scale bar, 100 μm, error bars SEM (ELISAs *n* = 3; technical replicates = 2, cell culture experiments *n* = 4; technical replicates = 3)

Amyloid beta A4 protein (APP) is the precursor protein with many cleavage products including the Aβ 1–42 peptide and Aβ 1–40 peptide. These are the two major C‐terminal variants of the Aβ protein constituting the majority of Aβ peptides and undergo postsecretory aggregation and deposition in the Alzheimer's disease (AD) brain. To investigate the presence of these peptides and validate the TMT‐LC/MS analysis, an ELISA for the 1–42 peptide and 1–40 peptide was carried out on the eluate from the mHep7 column (Figure 8a,b). This demonstrated that there was a significant increase in concentration for both Aβ peptides in the DCM eluate compared to UCM. However, this increase was not observed in the CCM eluate, suggesting that the increased abundance for the APP hit in CCM was due to a different cleavage product. This suggests that the APP cleavage product in the CCM mHep eluate may be from the nonamyloidogenic pathway. Moreover, the sequence of peptide fragment identified in the mass spectrometry analysis is a 12 residue peptide corresponding to residues 439–450 of the precursor protein and represents the soluble product of the initial cleavage by either α‐secretase (nonamyloidogenic pathway) or β‐secretase (amyloidogenic pathway); therefore, the peptide can be from either pathway.

### 3.10 mHep7 treatment modulates Aβ peptide concentration following demyelination

To confirm the exclusive increase of amyloidogenic peptides in MC‐Demy, ELISAs were performed on the UCM, CCM, and DCM (D0). This demonstrated a sole increase in the DCM of both Aβ 1–42 (4‐fold) and Aβ 1–40 (42‐fold) compared to the UCM (Figure 8c,d; *p* = .0007 and .0017). As expected we observed a greater abundance of Aβ1–40 compared with Aβ1–42. This is because Aβ1–40 makes up a larger proportion of Aβ peptides with the average ratio of 1–40:1–42 being 10:1 (Eckman et al., 1997). There was a large increase in Aβ1–40 levels immediately after demyelination (D0, Figure 8d) however 24 hr later (D1, Figure 8f) this had dropped around 10‐fold, we speculate this is due to the short half‐life of this peptide (Farris et al., 2007). To establish the effect of mHep treatment on Aβ 1–42 and Aβ 1–40 concentration, MC‐Demy were treated with mHep7 and CM collected at days 1 (D1) and 3 (D3) posttreatment. There was a significant increase in Aβ 1–42 (1.8‐fold) and Aβ 1–40 (9‐fold) peptides concentration at D1 in the CM following mHep7 treatment compared to the untreated controls (Figure 8e,f; *p* = .0003 and .001, respectively). However, by D3, there seems to be a significant decrease in Aβ 1–42 (0.67‐fold) and Aβ 1–40 (0.125‐fold) peptides concentration compared to the untreated control (*p* = .0096 and .0001, respectively). The results suggest that treatment with mHep7 can modulate the level of Aβ peptides present in the CM.

### 3.11 mHep treatment can overcome Aβ (1–42) inhibition of myelination

To examine whether Aβ 1–42 peptide has any adverse effect on developmental myelination, an MC‐Dev time course was treated with Aβ 1–42 peptide (with co‐treatment of mHep7). CM was collected and used in a cytotoxicity assay to establish if treatments induced cell death. Treatment of MC‐Dev cultures with Aβ 1–42 peptide resulted in a 49% reduction in myelination at 24 DIV (Figure 8g–j; *p* = .0082). The absence of any effect on neurite density following treatment along with the cytotoxicity assay demonstrated that this was not due to generic cell death (Figure 8k). This inhibitory effect could be rescued by co‐treatment with mHep7 (*p* = .032). The ELISA data suggests that mHep7 binds both Aβ1–40 and Aβ1–42 peptides reducing levels present in the cell culture medium after demyelination. Thus, we hypothesize that mHeps bind to and sequester Aβ1–42 in culture, preventing its mode of action and so rescuing its inhibition of myelination.

## 4 DISCUSSION

### 4.1 Cellular targets for HS: Astrocytes and neurons

Treatment with LS‐mHeps showed multiple beneficial outcomes in our MC‐Inj cultures by promoting myelination, increasing neurite density and outgrowth, and decreasing lesion size. This suggests these compounds are acting on either multiple cellular targets or targeting a specific cell type which in turn can mediate several different cellular processes, such as the astrocyte. After CNS injury, astrocytes become reactive and secrete inflammatory molecules that modify the environment around the injury or disease (Barnett & Linington, 2013; Hara et al., 2017; O'Shea, Burda, & Sofroniew, 2017; Williams, Piaton, & Lubetzki, 2007). Moreover, after an injury to the adult rat brain, there is an overall increase in the quantity of HSPG around the injury site, as well as an increase in mRNA for heparan 2‐O‐sulfotransferase (HS2ST) and subsequently the level of 2‐O‐sulfated HS (Properzi et al., 2008). Nevertheless, we found little evidence of reactivity induction following mHep treatment in scratch astrocyte assays or after Western blotting with GFAP (data not shown). In addition, changes in sulfation patterns of HSPGs have many effects on axon growth and guidance. For example, HS2ST and heparan 6‐O‐sulfotransferase (HS6ST) knockout mice have shown multiple navigational errors in their axons, probably due to disturbance of the guidance effects of slit proteins (Pratt, Conway, Tian, Price, & Mason, 2006), and genetic manipulations of syndecan expression in Drosophila showed similar guidance defects (Johnson et al., 2004). Although information is known about the role of HSPGs in the development of the CNS, their function in nerve injury is not yet fully understood (Murakami, Tanaka, Bando, & Yoshida, 2015). There have been some reports of upregulation of syndecan‐1 and glypicans after injury (Leadbeater et al., 2006) and evidence that this promotes neurite outgrowth but the mechanism is not known. It is likely that the HS chains carried by these HSPGs could be targets for the LS‐mHeps.

Our data suggest that the sulfation level or its position on the HS disaccharide is crucial in regulating cellular function. Using our panel of mimetics, we found that only the monosulfated forms at the 2‐O‐ and N‐sulfated positions promoted neurite outgrowth. This is in agreement with a study that used mutants of the Hst‐2 gene thereby reducing 2‐O‐sulfation on the HS leading to axonal patterning defects (Kinnunen, Townsend, & Turnbull, 2004). These experiments suggest that the 2‐O‐sulfate moiety is involved in neurite outgrowth and pathfinding. However, LS‐mHep8 which lacks the 2‐O‐ and 6‐O‐sulfate also promotes neurite outgrowth, implying that the reduced sulfation level of these mHeps may be contributing to the observed outgrowth. Others have shown that applying HS with different sulfation modifications disrupts axons guidance in the Xenopus visual system, with 2‐O‐ and 6‐O‐sulfated HS having the most marked effects (Irie, Yates, Turnbull, & Holt, 2002). Therefore, it has been postulated that there is a sulfation code that regulates axon guidance (Holt & Dickson, 2005). The LS‐mHeps could be directly affecting neurite outgrowth, and astrocytosis through artificially mimicking their interaction with the growing neurite (Lander, Fujii, Gospodarowicz, & Reichardt, 1982), or indirectly by altering surrounding cellular behavior, creating a permissive environment for outgrowth and re/myelination.

### 4.2 HS role in re/myelination

We originally considered that mHeps might regulate growth factors which act in concert to drive efficient myelination of OPCs. However, there was no effect of their treatment in developmental myelination occurring only when cultures were injured. We hypothesise this is because endogenous HS are sufficient for developmental myelination but during injury, there are dramatic changes in the extracellular environment suggesting that the LS‐mHeps may elicit their effects through interacting with secreted factors present in this aberrant injury environment.

### 4.3 Identification of mHep binding‐proteins in CM from MC‐Inj and MC‐Demy

To examine the molecular basis for the pro‐repair effects in postinjury myelinating cultures we aimed to identify heparin‐binding proteins in two ways. First, we examined chemokine/cytokine candidates, and secondly conducted TMT‐LC/MS. The chemokine/cytokine array illustrated major differences in the secretome between the MC‐Demy and MC‐Inj. In the former immune‐mediated MC‐Demy, inflammatory factors in DCM were more prominent than in CCM. This suggests that these two different culture conditions affect remyelination by distinct mechanisms. For example, IL1α, TNF‐α, and C1q (found in DCM, see Figure 6) induce the neurotoxic A1 astrocyte phenotype (Liddelow et al., 2017) suggesting that in MC‐Demy this astrocyte phenotype may be affecting remyelination, as previously reported (Nash et al., 2011). Thus, mHeps may be removing or inhibiting A1 astrocyte inducing factors, therefore promoting remyelination.

Our initial MS analysis was nonquantitative but strongly indicated pull down heparin‐binding proteins as the most significant group proteins (GO enrichment). GO term analysis (data not shown) confirmed CCM contained many factors involved in neurite outgrowth, guidance migration, and changes in astrocyte development. In contrast, DCM contained factors that were related to chemokine signaling and the immune system. Subsequent TMT LC–MS analysis yielded fold changes relative to the uninjured control (UCM) and allowed us to perform direct comparisons between the different CMs with certainty.

The TMT LC–MS data analysis identified a smaller panel of factors but with quantitative data from samples analyzed in duplicate. The reduced sensitivity is a likely consequence of the multiplexing approach, but the focus on proteins of a higher abundance may remove outliers and aid in narrowing our list of candidates. As seen with the cytokine array there was increased expression of immune‐mediated factors in DCM. The reduced list of hits enabled a literature search focused on secreted proteins with relevance to CNS injury.

### 4.4 Factors found in CCM

Table 2a illustrates candidates isolated from CCM that were expressed at greater than 1.3‐fold increase when compared to UCM. A2M, a broad spectrum proteinase inhibitor and a carrier of growth factors was increased in CCM. It has neuromodulatory activities (Wolf & Gonias, 1994) and has been demonstrated as a marker of neuronal injury and associated with preclinical AD (Varma et al., 2017). Apolipoprotein D (ApoD) is a secreted glycoprotein with many roles within lipid transport, detected in neurons, astrocytes, and oligodendrocytes (Ong et al., 1999). It has been associated with neurological disorders (multiple sclerosis [MS] and AD), other inflammatory diseases of the CNS and nerve injury (Li et al., 2015; Reindl et al., 2001). Interestingly levels were not above threshold in MC‐Demy, suggesting a nerve damage component to its upregulated expression.

Clusterin (CLU) a small heat shock protein that can act as a molecular chaperone protein was also upregulated. CLU like ApoD, has been implicated in ameliorating oxidative stress in neurodegenerative diseases and may be involved in the death of damaged neurons (Törnqvist, Liu, Aldskogius, Holst, & Svensson, 1996). It has been identified in the cerebrospinal fluid (CSF) of patients with MS (van Luijn et al., 2016) and AD (Matsuoka et al., 2001; Wojtas et al., 2017) and thought to act as a carrier of several proteins across the BBB and CSF barrier including amyloid‐β (Aβ) (Ghiso et al., 1993; Zlokovic et al., 1996). It has been suggested that CLU directly interacts with Aβ, thereby regulating its clearance from the brain (Bell et al., 2007). Amyloid precursor protein (APP), which was also secreted in CCM and DCM, is known to be upregulated during axonal injury in MS (Ferguson, Matyszak, Esiri, & Perry, 1997), and CLU may clear it and thereby prevent aggregation of Aβ. It was also interesting that RAGE was upregulated in the cytokine array as it is thought to play a role in the peripheral re‐entry of Aβ into the brain (Deane et al., 2003).

### 4.5 Factors found in DCM

Table 2b shows there were more candidates pulled down exclusively in DCM compared to CCM. Several have already been implicated in CNS injury, for example tenascin C (TnC), a glycoprotein synthesized by astrocytes and secreted into the ECM. Increased expression of TnC has been implicated after demyelination in vivo (Zhao, Fancy, Franklin, & ffrench‐Constant, 2009) and been shown to inhibit OPC differentiation in vitro both directly and indirectly through astrocytes (Czopka, von Holst, ffrench‐Constant, & Faissner, 2010; Nash et al., 2011). Moreover, knockout of TnC resulted in a favorable outcome on ADs pathology in vivo (Xie et al., 2013). Another ECM glycoprotein thrombospondin 1 (TSP‐1) also appeared to have increased abundance in DCM. TSP‐1 interacts with Neuroligin 1 to accelerate synaptogenesis of hippocampal neurons (Xu, Xiao, & Xia, 2010) but also reported to promote OPC migration (Scott‐Drew & ffrench‐Constant, 1997).

Gelsolin, an actin regulatory factor was identified in DCM. Mice lacking gelsolin, display a delayed remyelination after PNS crush injuries, presumed due to gelsolin recruiting macrophages to the injury site (Gonçalves et al., 2010). Moreover, gelsolin knockout mice had wrapping defects in the CNS, (Zuchero et al., 2015). This implies gelsolin might have a dual role after injury, firstly through the recruitment of immune cells to clear debris and secondly facilitating oligodendrocyte axon ensheathment.

### 4.6 Candidate factors identified in both DCM and CCM

In Table 2c, it can be seen that a few candidates were upregulated in both CCM and DCM and therefore may relate to promoting myelination. For example, growth hormone releasing hormone receptor has been detected in the CNS, and its ligand activity promotes the secretion of insulin‐like growth factor (IGF‐1, Zhao et al., 2008), a known mitogen for OPCs and an important regulator of brain development, maintenance and neurogenesis (Aberg, 2010). Moreover, APP was identified in both CMs. HS has been shown to interact with Aβ peptides and thus been implicated in facilitating Aβ cytotoxicity and accelerating amyloid fibril formation (Castillo, Ngo, Cummings, Wight, & Snow, 1997; Sandwall et al., 2010). Additionally, infusion with Aβ and HS into rat brains resulted in increased amyloidosis compared to Aβ alone (Snow, Sekiguchi, Nochlin, Kalaria, & Kimata, 1994). This suggests that endogenous HS somewhat aids Aβ aggregation pathology. Aβ has been shown to be secreted in excess following traumatic injury (Gentleman, Nash, Sweeting, Graham, & Roberts, 1993) and MS lesions (Pajoohesh‐Ganji et al., 2014). Although not understood in these pathological conditions Aβ has been reported to have negative correlation with functional outcome and induce microglia activation, inflammation, and neuronal cell death (Matsuoka et al., 2001).

Due to the detection of Aβ in MC‐Inj and MC‐Demy and its known role in CNS pathology we decided to focus more on its function and interaction with mHeps in these cultures. Since APP has numerous different fragment peptides, it was important to determine the peptides identified in the TMT/LC–MS. ELISAs of the mHep7 eluates and CMs suggests that after demyelination in MC‐Demy Aβ peptides (1–40/1–42) are secreted, and that the degradation of these peptides is modulated directly by mHep treatment. Previously, it has been demonstrated that treatment with Aβ 1–42 inhibits OPC differentiation (Horiuchi et al., 2012), in this study, we developed this further showing that the effect was present at the level of myelination and could be rescued through mHep co‐treatment. However, it appears that the APP detected in the CCM mHep7 eluate TMT‐LC analysis was not Aβ 1–40 or 1–42 peptides, suggesting it was the P3 peptide from the nonamyloidgenic pathway. This peptide is the equivalent of Aβ 17–40/42, although this fragment does not assemble into soluble oligomers, it does possess cellular toxic properties (Dulin et al., 2008; Wei, Norton, Wang, & Kusiak, 2002). Hence the P3 peptide could be a mHep7 modulated negative injury factor secreted after MC‐Inj. As Aβ peptides have been reported in both neurodegeneration and traumatic injury, with studies demonstrating the therapeutic benefits of targeting BACE‐1 in AD (Scholefield et al., 2003). This Aβ peptides could be a valuable target for mHep7 and the action of sequestering or inhibiting aggregation, could promote neurite outgrowth and myelination in MC‐Inj and MC‐Demy.

In summary, the present study has demonstrated beneficial effects of LS‐mHeps on repair in models of CNS damage specifically promoting neurite outgrowth and myelination by modifying the properties of secreted factors generated after injury. Our data has identified multiple protein candidates for mediating these effects and thus plausible underlying mechanisms. Moreover, this illustrates the complexity of mediating repair and highlights that therapeutics need to target many factors, as seen by LS‐mHeps. Furthermore, we show Aβ peptides can play a role in demyelination. Finally, based on the protein hits and the relation of some of these proteins to other neurological disease such as AD, these novel compounds could also have therapeutic potential in other neurological disorders.

## CONFLICT OF INTEREST

The authors declare no competing financial interests.
